# Evaluation of long-term stability and relapse rates in open apex teeth following surgical de-rotation and splinting

**DOI:** 10.6026/9732063002001349

**Published:** 2024-10-31

**Authors:** Avijit Das, Kuldeep Pal, Poorvi Saxena, SK Naja Parveen, Sachin Kumar Jaiswal, Shalini Singh

**Affiliations:** 1 Department of Orthodontics & Dentofacial Orthopaedics, North Bengal Dental College & Hospital, Sushruta Nagar, West Bengal, India; 2 Oral & Maxillofacial Surgery, Consultant Maxillofacial Surgeon at Pal Hospital, Sagar, Madhya Pradesh, India; 3 Department of Conservative Dentistry and Endodontics, Geetanjali Dental and Research Institute, Udaipur, Rajasthan, India; 4 Department of Paediatric and Preventive Dentistry, Dental College Azamgarh, Ghazipur, Azamgarh, Uttar Pradesh, India; 5 Department of Orthodontics & Dentofacial Orthopaedics, Dental College Azamgarh, Ghazipur, Azamgarh, Uttar Pradesh, India; 6 Department of Pedodontics and Preventive Dentistry, School of Dental Science, Sharda University, Greater Noida, Uttar Pradesh, India

**Keywords:** Open apex teeth, surgical de-rotation, splinting, long-term stability, relapse rates, periodontal health, dental alignment

## Abstract

The management of open apex teeth requiring surgical intervention poses a significant challenge in dental practice, particularly concerning the long-term stability and potential relapse following treatment. Surgical de-rotation and splinting are common procedures used to correct misalignment in such teeth. This study aims to evaluate the long-term stability and relapse rates in open apex teeth following surgical de-rotation and splinting over a two-year follow-up period. A prospective cohort study was conducted involving 30 patients aged 10-15 years with open apex teeth requiring surgical de-rotation. Each patient underwent surgical de-rotation followed by splinting for a period of six weeks. The patients were monitored at 6 months, 12 months, and 24 months post-treatment. Clinical parameters such as tooth alignment, periodontal health and any signs of relapse were assessed using radiographic and clinical examination techniques. Data were analyzed using descriptive statistics and the chi-square test to evaluate relapse rates. Out of the 30 patients treated, 28 (93.3%) showed satisfactory alignment and periodontal health at the 6-month follow-up. At 12 months, 26 patients (86.7%) maintained stability, while 4 (13.3%) exhibited minor relapse. By the 24-month follow-up, 22 patients (73.3%) demonstrated long-term stability, while 8 patients (26.7%) experienced relapse. The chi-square test indicated a statistically significant increase in relapse rates over time (p < 0.05). No significant differences were observed in periodontal health between stable and relapsed cases. Surgical de-rotation and splinting of open apex teeth demonstrate a high initial success rate, but relapse rates increase significantly over time. Continuous monitoring and potential adjunctive therapies may be necessary to maintain long-term stability. These findings highlight the importance of patient-specific treatment planning and long-term follow-up in managing open apex teeth.

## Background:

The treatment of open apex teeth presents a significant challenge in dental practice, particularly when it comes to ensuring long-term stability following corrective procedures. Open apex teeth often arise from developmental disturbances, trauma or carious exposure, leading to complications such as incomplete root formation and increased susceptibility to misalignment [1, 2]. The traditional approach to managing such teeth involves endodontic techniques aimed at promoting apexification or apical closure [3]. However, cases requiring orthodontic correction due to mal-alignment necessitate additional interventions, such as surgical de-rotation and splinting. Surgical de-rotation is a technique used to correct the rotational malposition of teeth, often combined with splinting to stabilize the corrected position [4]. The splint serves to support the tooth during the healing process and to maintain alignment, allowing for periodontal ligament adaptation and functional integration [5]. Despite the initial success of these interventions, the long-term stability of treated open apex teeth remains a concern, with relapse being a common issue observed in clinical practice [6]. The factors contributing to relapse include insufficient periodontal support, inadequate healing of the periodontal ligament, and external forces acting on the teeth, such as occlusal stress and muscular activity [7]. Previous studies have indicated that the risk of relapse increases over time, highlighting the need for long-term follow-up and potentially adjunctive therapies to maintain treatment outcomes [8, 9]. The objective of this study is to evaluate the long-term stability and relapse rates in open apex teeth following surgical de-rotation and splinting over a two-year period. By analyzing clinical outcomes and identifying predictors of relapse, this study aims to contribute to the optimization of treatment strategies for open apex teeth, ultimately improving patient care and treatment efficacy.

## Materials and Methods:

## Study design and setting:

This prospective cohort study was conducted at the Department of Periodontology, over a period of 24 months.

## Participants:

A total of 30 patients, aged 10 to 15 years, with open apex teeth requiring surgical de-rotation were included in the study. The inclusion criteria were: (1) presence of open apex permanent teeth with mal alignment, (2) absence of systemic health issues and (3) willingness to comply with follow-up visits. Patients with significant periodontal disease, previous orthodontic treatment, or incomplete records were excluded.

## Intervention:

## Surgical de-rotation and splinting:

Each patient underwent surgical de-rotation of the affected open apex teeth. The procedure was performed under local anesthesia, involving the careful rotation of the tooth to its ideal position. Following de-rotation, a semi-rigid splint was applied using a composite resin and orthodontic wire to stabilize the tooth for six weeks. Post-operative care included instructions on oral hygiene and dietary restrictions to minimize stress on the splinted teeth.

## Follow-Up and outcome measures:

Patients were followed up at intervals of 6 months, 12 months and 24 months post-treatment. The primary outcome measures were the stability of the tooth alignment and relapse rates. Stability was assessed through clinical examination and radiographic analysis using standardized periapical radiographs. Relapse was defined as a clinically significant return to the original malposition, measured by comparing post-treatment and follow-up alignments. Periodontal health was also evaluated at each follow-up visit by assessing gingival condition, probing depth and attachment level. All examinations were conducted by a single calibrated examiner to ensure consistency and reliability of the measurements.

## Statistical analysis:

Data were analyzed using SPSS software (version 25.0; IBM Corp., Armonk, NY, USA). Descriptive statistics were used to summarize demographic and clinical characteristics. The chi-square test was employed to assess the association between relapse rates and time. A p-value of < 0.05 was considered statistically significant.

Overall, the periodontal health of all patients remained within clinically acceptable limits throughout the study period, with no significant deterioration observed.

## Patient satisfaction:

Patient satisfaction was assessed using a questionnaire at the end of the 24-month period. Results indicated that 90% of patients (27 out of 30) were satisfied with the esthetic and functional outcomes of the treatment, despite the occurrence of relapse in some cases.

## Results:

A total of 30 patients were enrolled in the study, with a mean age of 12.5 ± 1.8 years. Of these, 18 were males and 12 were females. All patients completed the 24-month follow-up. The outcomes of surgical de-rotation and splinting were assessed in terms of stability and relapse rates at various intervals.

## Stability and relapse rates:

The stability and relapse rates of the treated teeth were evaluated at 6 months, 12 months and 24 months post-treatment. The initial success rate at 6 months was high, with 28 out of 30 patients (93.3%) maintaining satisfactory alignment. However, by 12 months, 4 patients (13.3%) experienced minor relapse and at the 24-month follow-up, the number of patients with relapse increased to 8 (26.7%) (Table 1).

The chi-square test revealed a statistically significant increase in relapse rates over time (p = 0.03), indicating that the likelihood of relapse increases as time progresses.

## Periodontal health:

The periodontal health of patients was assessed using gingival condition, probing depth and attachment level. The results showed no significant differences in periodontal health between patients with stable alignment and those who experienced relapse at each follow-up interval (Table 2).

## Discussion:

The management of open apex teeth presents a unique challenge due to the delicate nature of their root development and the tendency for misalignment. This study evaluated the long-term stability and relapse rates following surgical de-rotation and splinting of open apex teeth over a two-year period. The findings indicate a high initial success rate, with 93.3% of patients maintaining stable alignment at 6 months post-treatment. However, the relapse rate increased significantly to 26.7% by the 24-month follow-up, underscoring the challenges in maintaining long-term stability. Our findings are consistent with previous studies that have reported similar challenges in managing open apex teeth. Marending *et al.* [1] highlighted the difficulties in achieving predictable outcomes with endodontic treatments in immature teeth, which often require additional interventions to address misalignment. The increase in relapse rates observed in this study is consistent with the literature, which suggests that factors such as inadequate periodontal support and external forces can contribute to relapse over time [2- 3]. The use of splinting in conjunction with surgical de-rotation has been shown to be effective in maintaining initial tooth alignment. Uribe and Nanda [4] demonstrated the importance of splinting in stabilizing teeth post-orthodontic or surgical intervention, allowing for proper healing and adaptation of the periodontal ligament. However, the long-term efficacy of splinting remains a topic of debate, as the presence of a semi-rigid splint may not fully prevent relapse, particularly in the presence of occlusal forces [5]. The periodontal health of patients in this study remained stable throughout the follow-up period, with no significant differences observed between those who maintained stable alignment and those who experienced relapse. This finding aligns with Artun and Urbye [6], who reported that orthodontic interventions, when combined with proper oral hygiene measures, do not adversely affect periodontal health. Nonetheless, the potential impact of orthodontic and surgical procedures on the periodontium requires careful monitoring, as emphasized by Littlewood *et al.* [7]. The patient satisfaction rate in this study was high, with 90% of participants expressing satisfaction with the esthetic and functional outcomes. This aligns with Johnston *et al.* [8], who emphasized the importance of patient perceptions in evaluating the success of orthodontic treatments. Despite the occurrence of relapse in some cases, the overall positive response suggests that patients prioritize visible improvements in alignment and aesthetics. The limitations of this study include the relatively small sample size and the lack of control over variables such as occlusal forces and individual healing responses, which may influence relapse rates. Future research should focus on larger cohorts and explore adjunctive therapies or interventions that could enhance long-term stability, as suggested by Proffit *et al.* [9].

## Conclusion:

In conclusion, while surgical de-rotation and splinting are effective in achieving initial alignment of open apex teeth, maintaining long-term stability remains a challenge. Continuous monitoring and patient-specific treatment plans are essential to optimize outcomes. The findings of this study contribute to the understanding of relapse dynamics and highlight the need for on-going research in this area.

## Figures and Tables

**Table 1:** Stability and relapse rates over time

**Follow-Up Period**	**Number of Patients**	**Stable Alignment (%)**	**Relapse (%)**
6 Months	30	28 (93.3)	2 (6.7)
12 Months	30	26 (86.7)	4 (13.3)
24 Months	30	22 (73.3)	8 (26.7)

**Table 2:** Periodontal health assessment

**Follow-Up Period**	**Mean Probing Depth (mm)**	**Mean Attachment Level (mm)**	**Gingival Health Score (1-5)**
6 Months	2.1 ± 0.4	0.5 ± 0.2	1.2 ± 0.3
12 Months	2.3 ± 0.5	0.6 ± 0.3	1.4 ± 0.4
24 Months	2.4 ± 0.5	0.7 ± 0.3	1.5 ± 0.4
